# Initial Experience with the Solitaire X 3 mm Stent Retriever for the Treatment of Distal Medium Vessel Occlusions

**DOI:** 10.3390/jcm12237289

**Published:** 2023-11-24

**Authors:** Nikos Ntoulias, Alex Brehm, Ioannis Tsogkas, Jessica Jesser, Antonio Armando Caragliano, Theo Demerath, A. C. G. M. van Es, Phillip Gruber, Pedro Vega, Alex Lüttich, Sanjeev Nayak, Eduardo Fandiño, Marc Ribo, Carlos Manuel Rodriguez Paz, Markus A. Möhlenbruch, Agostino Tessitore, Luca Remonda, Eduardo Murias, Kristine Ann Blackham, Marios-Nikos Psychogios

**Affiliations:** 1 Department of Interventional and Diagnostic Neuroradiology, University Hospital of Basel, 4031 Basel, Switzerland; nikolaos.ntoulias@usb.ch (N.N.); alex.brehm@usb.ch (A.B.); ioannis.tsogkas@usb.ch (I.T.); kristineann.blackham@usb.ch (K.A.B.); 2 Department of Neuroradiology, Heidelberg University Hospital, 69120 Heidelberg, Germany; jenni.jesser@med.uni-heidelberg.de (J.J.); markus.moehlenbruch@med.uni-heidelberg.de (M.A.M.); 3 Neuroradiology Unit, University Hospital “G. Martino”, 98124 Messina, Italy; caraglia1987@gmail.com (A.A.C.); agostinotessitore@gmail.com (A.T.); 4 Neuroradiologie Neurozentrum, Universitätsklinikum Freiburg, 79106 Freiburg, Germany; 5 Department of Radiology, Leiden University Medical Center, 2333 ZA Leiden, The Netherlands; a.c.g.m.van_es@lumc.nl; 6 Faculty of Medicine, University of Zürich, 8006 Zürich, Switzerland; phillip.gruber@ksa.ch; 7 Department of Diagnostic and Interventional Neuroradiology, Kantonsspital Aarau, 5001 Aarau, Switzerland; luca.remonda@ksa.ch; 8 Radiology Department, Hospital Universitario Central de Asturias, 36312 Vigo, Spain; peveval@yahoo.es (P.V.);; 9 Interventional Neuroradiology, University Hosptal Donostia, 20014 Donostia, Spain; luttich21@hotmail.com; 10 Department of Interventional Neuroradiology, Royal Stoke University Hospital, University Hospital of North Midlands, Stoke-on-Trent ST4 6QG, UK; sanjeev.nayak@uhnm.nhs.uk; 11 Hospital Universitario Ramon y Cajal, 28034 Madrid, Spain; fandieduar@hotmail.com; 12 Stroke Unit, Neurology Department, Hospital Vall d‘Hebron, 08035 Barcelona, Spain; marcriboj@hotmail.com; 13 Hospital Alvaro Cunqueiro, 36312 Vigo, Spain; carlos.manuel.rodriguez.paz@sergas.es; 14 Faculty of Medicine, University of Bern, 3012 Bern, Switzerland

**Keywords:** stroke, endovascular thrombectomy, distal medium vessel occlusions

## Abstract

Endovascular therapy (EVT) is the standard treatment for ischemic stroke caused by a large vessel occlusion (LVO). The effectiveness of EVT for distal medium vessel occlusions (MDVOs) is still uncertain, but newer, smaller devices show potential for EVT in MDVOs. The new Solitaire X 3 mm device offers a treatment option for MDVOs. Our study encompassed consecutive cases of primary and secondary MDVOs treated with the Solitaire X 3 mm stent-retriever as first-line EVT device between January and December 2022 at 12 European stroke centers. The primary endpoint was a first-pass near-complete or complete reperfusion, defined as a modified treatment in cerebral infarction (mTICI) score of 2c/3. Additionally, we examined reperfusion results, National Institutes of Health Stroke Scale (NIHSS) scores at 24 h and discharge, device malfunctions, complications and procedural technical parameters. Sixty-eight patients (38 women, mean age 72 ± 14 years) were included in our study. Median NIHSS at admission was 11 (IQR 6–16). In 53 (78%) cases, a primary combined approach was used as the frontline technique. Among all enrolled patients, first-pass mTICI 2c/3 was achieved in 22 (32%) and final mTICI 2c/3 in 46 (67.6%) patients after a median of 1.5 (IQR 1–2) passes. Final reperfusion mTICI 2b/3 was observed in 89.7% of our cases. We observed no device malfunctions. Median NIHSS at discharge was 2 (IQR 0–4), and no symptomatic intracranial hemorrhages were reported. Based on our analysis, the utilization of the Solitaire X 3 mm device appears to be both effective and safe for performing EVT in cases of MDVO stroke.

## 1. Introduction

Endovascular therapy (EVT) has become the standard treatment for acute ischemic stroke (AIS) due to a large vessel occlusion (LVO) of the anterior circulation and basilar artery after evidence from randomized controlled trials (RCTs) demonstrated clear benefits of EVT [1,2,3,4,5,6]. For distal medium vessel occlusions (MDVOs), the indication to perform EVT is less clear; multiple RCTs are actively enrolling patients to address this important unanswered clinical question (DISTAL (NCT05029414), DISTALS (NCT05152524), DISCOUNT (NCT05030142) and ESCAPE-MeVO (NCT05151172)). Newer and smaller devices might be beneficial in performing EVT in MeVOs [7,8]. Retrospective studies suggest good recanalization rates and adequate safety [9,10].

The Solitaire platform is a well-established stent retriever (SR) which has shown its efficacy in multiple RCTs [3,5,11]. Recently, the new Solitaire X 3 mm was introduced as a new device for the treatment of MDVOs which occur on vessels often smaller in diameter than those of LVOs. According to the manufacturer, the device can be safely deployed in vessels with a diameter as small as 1.5 mm. However, no data on its efficacy and safety for the treatment of such occlusions have been published so far.

In this retrospective study, we assessed the new 3 mm Solitaire X SR for the treatment of AIS due to MDVOs. The main endpoints were reperfusion results as well as peri- and post-interventional complications and clinical outcomes.

## 2. Materials and Methods

We collected consecutive cases from 12 European EVT centers from January to December 2022. Inclusion criteria were (a) the use of the Solitaire^TM^ X 3 mm (Medtronic, Irvine, CA, USA) SR as the frontline EVT device for treatment and (b) a stroke due to an MDVO. The only exclusion criteria were patient age <18 years and refusal of research consent. This study was approved by the applicable ethics committee. 

Data were extracted from institutional databases regarding the baseline clinical status of patients; National Institutes of Health Stroke Scale (NIHSS) at baseline, at 24 h post-procedure and at discharge; and modified Rankin Scale (mRS) pre- and post- stroke, assessed by certified stroke neurologists of each participating center. 

Qualifying imaging was performed according to each participating center‘s protocols and assessed by experienced radiologists from each center for Alberta stroke program early CT score (ASPECTS), perfusion parameters and tandem or secondary occlusions.

Angiography series were assessed by the performing interventionalist. Post-interventional imaging was evaluated for intracranial hemorrhage, contrast medium extravasations or hemorrhagic transformation of the infarct according to the European Cooperative Acute Stroke Study-2 (ECASS-2) criteria. Symptomatic intracranial hemorrhage (sICH) was defined as an intracranial hemorrhage that was associated with clinical deterioration, as documented by an increase of ≥4 points on the NIHSS. In intubated patients, sICH was defined by the ECASS-2 criteria as any parenchymal hematoma grade I or II.

We also looked into technical and anatomic parameters in a per-pass approach, evaluating this stent retriever in conjunction with other EVT devices.

EVT procedures were performed based on the standard of practice of each participating center. The two eligible techniques were (a) the primary combined approach (PCA): Solitaire X 3 mm SR in combination with aspiration catheter (AC) or (b) SR-alone: Solitaire X 3 mm with the use of a balloon guide catheter (BGC) or a long sheath. 

The dataset, after the data acquisition phase of the study was concluded, was processed by one of the investigators (N.N.). Statistical analysis was performed at the University Hospital of Basel, with the use of the statistical software SPSS 27.0.1.0 (IBM Corp, Armonk, NY, USA).

## 3. Results

In total, 68 patients met the inclusion and exclusion criteria. Participants’ baseline characteristics are illustrated in Table 1. The mean age was 72 years, and 38 patients (56%) were female. Median NIHSS at admission was 11 (IQR 6–16) and median pre-stroke mRS was 0 (IQR 0–1). The most frequently used EVT technique was the PCA, used in 53 cases (78%), whereas the SR alone technique was used in 15 cases (22%). The overwhelming majority (97%) of EVTs were performed using femoral access, with just one case starting with femoral but finishing via radial access. 

In forty-three (63%) cases, there was an occlusion of the M2 segment, of which twenty-eight (65%) were in the superior trunk, fourteen (32%) in the inferior trunk and one case (3%) occurred in an occluded middle branch of a middle cerebral artery (MCA) trifurcation. Moreover, six (9%) M3 cases were reported, five (7.4%) cases of A1-, P1- and P2-occlusion, respectively, and one (1.2%) case each of M4, A2, P3 and superior cerebellar artery (SCA) occlusion. 

Concerning effectiveness, the first-pass complete or near-complete reperfusion (mTICI 2c/3) rate was 32.3%, while the final-pass mTICI 2c/3 was 67.6%. The median number of passes was 1.5 (IQR 1–2). 

Regarding technical parameters, the vast majority of EVTs were performed using aspiration catheters (AC) with diameters of 5F (22 cases) or 6F (22 cases). The average duration of waiting after SR deployment was 2 min and 40 s and in 45.5% of cases, it was less than 1 min. Lastly, when performing the thrombectomy maneuver using the PCA, blind-exchange with mini pinning of the microcatheter was utilized in 10% of cases [12]. In seven cases, the recently published Quattro technique was used [13].

In terms of safety, we observed no malfunctions in the 147 procedural passes conducted using the Solitaire X 3 mm. In 14 patients, complications of the MT were documented; among these, nine cases of subarachnoid hemorrhage (SAH) or contrast medium extravasation were detected immediately after the intervention through flat-detector computer tomography (FDCT), but none of the SAH patients experienced subsequent neurological deterioration. Additionally, three instances of vasospasm occurred, but all were resolved with intraarterial administration of nimodipine. Furthermore, two patients experienced iatrogenic vessel perforation. Finally, we documented an embolus in new territory in one (1.5%) case.

**Figure 1:**
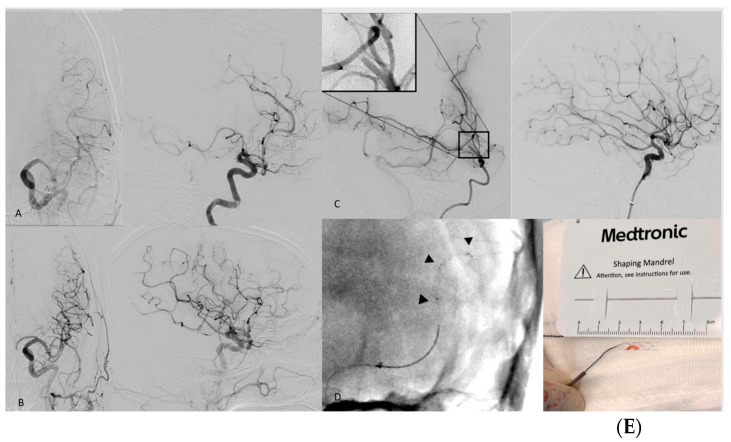
(**A**,**B**) Angiographic images of a 64-year-old stroke patient with an occlusion of the inferior trunk of the M2 segment on the left side; image B shows the result after recanalization eTICI 3. (**C**) M2 occlusion and deficit on lateral view of angiography with magnification of the occlusion. (**D**). 3D reconstruction using flat-detector CTA. (**C**) mTICI 3 result after successful EVT. (**D**) Highly visible Solitaire X 3 mm SR. (**E**) Ex-vivo photography of the Solitaire-X and a captured thrombus (Image curtesy of Dr. Lüttich).

Median NIHSS at 24 h post procedure was 6 (IQR 0–12) and it improved to 2 (IQR 0–7) at the time of discharge. All-cause in-hospital mortality was 10%. None of the deaths were deemed to be due to procedural complications (Figure 1, Table 2 and Table 3).

## 4. Discussion

Endovascular thrombectomy is the established treatment approach for patients with LVOs. This highly effective treatment is associated with favorable outcomes and high recanalization rates. While there is evidence supporting the expansion of the indications for EVT up to 24 h after onset and to the large core/infarct population, current evidence regarding the benefits of EVT for MDVOs is weaker [20,21]. Nevertheless, there is increasing interest in utilizing EVT for occlusions involving medium-sized and anatomically distal intracranial arteries, with patients currently being enrolled in multiple RCTs aiming to fill this evidence gap (DISTAL (NCT05029414), DISTALS (NCT05152524), DISCOUNT (NCT05030142) and ESCAPE-MeVO (NCT05151172)).

In our retrospective study, the goal was to assess the Solitaire X 3 mm for its effectiveness and safety in MDVO-EVT. The MDVOs in our cohort included anterior and posterior circulation strokes, with the great majority being M2 occlusions. Interestingly, we had more superior (65%) than inferior (32%) trunk occlusions, although superior trunk occlusions are less prevalent in the literature [22]. This difference could be attributed to the inferior trunk’s larger size and less tortuous course, which enables the treating physicians to deploy an SR larger than 3 mm, thus making these cases not eligible for the study.

Regarding reperfusion rates in our patient cohort, successful final reperfusion mTICI 2b/3 was 89.7% and complete/near-complete reperfusion mTICI 2c/3 was 67.6%. These results can be deemed improved when compared with the 77% mTICI 2b/3 found in recent systematic review on the topic, as well as when comparing our results with a similar-sized, SR-focused, MDVO-EVT retrospective series [23]. As thrombectomy is not the standard of care for MDVOs, only a few MDVO-dedicated SRs are commercially available and the retrospective design and limited number of cases in the published case series should be taken into account when comparing the Solitaire X 3 mm and its counterparts.

When we stratified our data according to the thrombectomy technique, the PCA was associated with improved recanalization rates for MDVO-EVT compared to the SR alone group. In our sub-group analysis, in the PCA group, TICI 2b/3 was achieved in 94%, in comparison to 91% in the SR alone group, while the percentage of final mTICI 2c/3 was 65% and 54.5%, respectively, in the PCA and SR alone groups. These results are in line with recent literature, where the PCA is more favorable than SR alone for MDVOs, with 83.7% and 75.6%, respectively [23]. Regarding NIHSS improvement from baseline to 24 h, the PCA group showed lower NIHSS values after 24 h, with a mean improvement of 5.29 (SD 6.54) vs. a mean of 4.45 (SD 5.92) for the SR alone group. Similarly, for NIHSS difference at discharge, the mean for the PCA group was 7.43 (SD 6.65) versus 4.00 (SD 12.30) for the SR alone group. However, it should be taken into account that the PCA group consisted of 53 patients while the SR alone group consisted only of 15. 

Another analyzed parameter was the FPE, which is associated with superior outcomes in LVO and MDVO thrombectomy. According to the literature, the FPE could be quantified, by technique, as 58% for PCA and 48% for SR alone in LVO-EVT, and in MDVO-EVT, as 55% for PCA and 46% for SR alone. In our MDVO group treated with Solitaire X 3 mm, FPE-mTICI 2c/3 was 34% for the PCA group and 27% for the SR alone group [24]. Moreover, considering the negative association of recanalization rate with an increased number of passes, the median 1.5 passes (IQR 1–2) found in our study can be deemed positive [25,26].

In relation to safety, the occurrence of peri-procedural SAH (13%) appears to have been higher in our cohort compared to data from LVO registries (5.6%), which can mainly be attributed to the inherent technical complexity of MDVO thrombectomy [27]. Treating occlusions in medium-sized vessels poses inherent challenges compared to treating LVOs. These challenges arise from the occlusions’ more distal location, increased vessel tortuosity, smaller diameter of the vessels through which thrombectomy materials must navigate and the susceptibility to injury of medium-sized arteries. Despite the increased technical complexity of the procedure and higher rate of SAHs, their clinical relevance seems to be low, as they did not manifest with accompanying clinical deterioration, as previously reported in the literature [28]. This can also be seen in our cohort, in which none of the nine SAHs was associated with neurological worsening. Our results present an acceptable safety profile that is comparable to other MDVO-dedicated SRs (see Table 3) and to the current literature, as the SAH rate in a study that assessed 1964 thrombectomies was 8.3% [23]. Another analyzed safety parameter was the frequency of peri-procedural perforation, a rare complication of EVT, which occurred in 1.3% of cases in a recent retrospective study assessing 25769 thrombectomies. Perforations are documented significantly more often in MDVO- than in LVO-EVT, with 2.4% versus 1% reported in the same study. Yet, functional outcomes after perforation are better in MDVO- than in LVO-EVT, a fact that is also depicted in our results, as one of the two patients experienced a clinical improvement and the other patient did not experience any clinical worsening despite the perforation [29]. Finally, the mortality rate of 10% in our more complex and technically challenging MDVO cohort is positive when compared to meta-analyzed Hermes data of an M2-occlusion-only population, in which the mortality rate of the EVT population was 12% [30]. 

To surpass the above-mentioned difficulty of the distal location of the occlusions, longer, smaller and softer materials (i.e., microcatheter, aspiration catheter) are required, ideally with good trackability which causes less stretching of the vessels, especially when performing a PCA because of the use of two aspiration catheters. 

One solution is the blind exchange of the microcatheter (BEMP) with a small aspiration catheter over the wire of the stent retriever. This method possesses the advantage of allowing a distal aspiration catheter to be placed exactly at the face of the clot, trapping it similarly to the SAVE technique [31]. On the other hand, BEMP has the disadvantage of the physician not always being in control of the SR while performing the exchange (thus “blind”). Blind exchange was utilized in 10% of our PCA cases. 

A possible solution to this shortcoming would be a quadriaxial approach to PCA in MDVO-EVT, as described in the QUATTRO technique [13]. In our cohort, the Quattro technique was used in seven patients, accounting for 13% of the PCA group. The advantages of this technique could mainly be summarized as (a) reducing the need for oversizing the aspiration catheter, thus lessening the danger of a dissection, (b) preventing vessel straightening and pulling of small perforators by creating more “joints” with the use of two aspiration catheters and (c) limiting the collapse of smaller arteries by starting the aspiration with the smaller aspiration catheter, only after a wedge position with the face of the clot is achieved [13].

### Limitations

Our study has the inherent limitations of retrospective studies (e.g., missing data) and studies of limited sample size. Also, baseline, interventional and post-interventional imaging were not rated by a central core lab, causing potential heterogeneity.

## 5. Conclusions

The new Solitaire X 3 mm is an effective and safe device for performing EVT in MDVOs, as its use is associated with good recanalization rates and an acceptable safety profile. A more thorough and in-depth analysis of its use with more parameters and in a larger patient group could provide us with important insight into this device. Also, in the future, data from currently recruiting RCTs will enable further study on the efficacy of this device, as well as on the optimization of its usage regarding combined EVT approaches to thrombectomy.

## Figures and Tables

**Table 1:** Baseline characteristics of the patients.

No. of Patients	68
Women, *n* (%)	38 (56%)
Age, mean ± SD	72 (58–86)
NIHSS at admission, median (IQR)	11 (6–16)
Pre-stroke mRS, median (IQR)	0 (0–1)
***Primary approach***	
Primary access	67 (98.5%) femoral; 1 (1.5%) radial
Final access	66 (97%) femoral; 2 (3%) radial
***Thrombectomy technique***	
Primary combined approach, *n* (%)	53 (78%)
Stent retriever alone, *n* (%)	15 (22%)
***Occlusion location***	
M2, *n* (%)	43 (63%)
M3, *n* (%)	6 (9%)
M4, *n* (%)	1 (1.5%)
A2, *n* (%)	5 (7.4%)
A3, *n* (%)	1 (1.2%)
P1, *n* (%)	5 (7.4%)
P2, *n* (%)	5 (7.4%)
P3, *n* (%)	1 (1.5%)
SCA, *n* (%)	1 (1.5%)
Intravenous thrombolysis, *n* (%)	27 (40%)
Secondary occlusions, *n* (%)	6 (9%)
Tandem occlusion, *n* (%)	5 (7%)
***Outcomes***	
NIHSS at 24 h, median (IQR)	6 (0–12)
NIHSS at discharge, median (IQR)	2 (0–7)
Subarachnoidal hemorrhage or contrast medium extravasation, *n* (%)	9 (15%)
**Safety**	
Emboli to new territory, *n* (%)	1 (1.5%)
Iatrogenic vessel perforation, *n* (%)	2 (3%)
*Any intracranial hemorrhage (on post-interventional imaging)*	
None, *n* (%)	52 (77%)
HI1, *n* (%)	7 (10%)
HI2, *n* (%)	1 (1.5%)
Subarachnoidal hemorrhage	9 (13%)
Worsening of ≥4 NIHSS points most likely related to hemorrhagic transformation, *n* (%)	0 (0%)
In-hospital mortality, *n* (%)	7 (10%)
**Waiting time in minutes, *n* (%)**	
0–1, *n* (%)	21 (31%)
2, *n* (%)	7 (10%)
3, *n* (%)	19 (28%)
4, *n* (%)	4 (6%)
5, *n* (%)	16 (23.5%)
Missing, *n* (%)	1 (1.5%)
**Aspiration catheter size (F)**	
3	7
4	8
5	22
6	22
**Anesthesia**	
Primary general anesthesia	34 (50%)
Conscious sedation	30 (44%)
Local anesthesia	4 (6%)

**Table 2:** Angiographic results stratified by occluded vessel and for all vessels.

	M2	M3	M4	A2	A3	P1	P2	P3	SCA
No. of patients	43	6	1	5	1	5	5	1	1
***First-pass reperfusion***									
mTICI ≥ 2b, *n* (%)	25 (58%)	3 (50%)	0	3 (60%)	1 (100%)	4 (80%)	2 (40%)		1 (100%)
mTICI ≥ 2c, *n* (%)	13 (30%)	2 (33%)	0	3 (60%)	1 (100%)	2 (40%)	0		1 (100%)
mTICI 3, *n* (%)	9 (21%)	1 (16.7%)	0	3 (60%)	0	1 (20%)	0		1(100%)
***Final reperfusion***									
mTICI ≥ 2b, *n* (%)	38 (88%)	6 (100%)	1 (100%)	4 (80%)	1 (100%)	5 (100%)	2 (40%)	3 (60%)	1 (100%)
mTICI ≥ 2c, *n* (%)	25 (58%)	4 (67%)	1(100%)	4 (80%)	1 (100%)	5 (100%)	2 (40%)	0	1 (100%)
mTICI 3, *n* (%)	13 (30%)	3 (50%)	1 (100%)	3 (60%)	1 (100%)	5 (100%)	0	0	1 (100%)

**Table 3:** Recanalization and subarachnoidal hemorrhage rates in other MeVO-dedicated SRs.

Study	Stent Retriever	% TICI ≥ 2b	% TICI ≥ 2c	Subarachnoidal Hemorrhage Rate (%)	Symptomatic Intracranial Hemorrhage Rate (%)
Fischer et al. 2022 [14]	Tigertriever 13	84.4%	-	14%	7%
Guenego et al. 2021 [15]	Tigertriever 13	94%	-	29%	-
Bernsen et al. 2021 [16]	Various	61%	41%	4%	-
Rikhtegar et al. 2021 [17]	Tigertriever 13	74.8%	-	-	6.9%
Perez-Garcia et al. 2020 [12]	Aperio 3.5/Catch mini	78.3%	56%	25.3%	6.6%
Hofmeister et al. 2018 [18]	Catch mini	78%	-	4.9%	0%
Müller-Eschner et al. 2018 [19]	Aperio 3.5	73.9%	-	4.5%	4.5%
**Our results**	**Solitaire X 3 mm**	**89.7%**	**67.6%**	**13%**	**0%**

## Data Availability

All data regarding this study are available upon request at nikolaos.ntoulias@usb.ch.
